# Effectiveness of a Protocol to Reduce Children’s Exposure to Particulate Matter and NO_2_ in Schools during Alert Days

**DOI:** 10.3390/ijerph191711019

**Published:** 2022-09-03

**Authors:** Stefano Zauli-Sajani, Stefano Marchesi, Giuseppe Boselli, Elisa Broglia, Alessandro Angella, Elena Maestri, Nelson Marmiroli, Annamaria Colacci

**Affiliations:** 1 Regional Agency for Prevention Environment and Energy of Emilia-Romagna (Arpae), 40139 Bologna, Italy; 2 Municipality of Parma, 43121 Parma, Italy; 3 Consorzio Interuniversitario Nazionale per le Scienze Ambientali (CINSA), Department of Chemistry, Life Sciences and Environmental Sustainability, University of Parma, 43124 Parma, Italy

**Keywords:** schools, indoor air quality, prevention, particulate matter, severe air pollution episodes

## Abstract

Reducing children’s exposure to air pollutants should be considered a primary goal, especially for the most vulnerable subjects. The goal of this study was to test the effectiveness of applying a protocol in the event of alert days, i.e., days with forecasted PM_10_ levels above the EU limit value (50 µg/m^3^). The test was conducted, before the onset of SARS-CoV-2 restrictions, in a classroom of a primary school in Parma (Italy)—a highly polluted area in Northern Italy. The protocol included indications for the frequency of opening windows and doors, as well as the activation of an air purifier. Teachers and students were asked to apply the protocol only in the event of alert days, while no indications were provided for non-alert days. A monitoring system measuring PM_1_, PM_2.5_, PM_10_, CO_2_, and NO_2_ was deployed in the classroom. Measurements of the same parameters were also performed outdoors near the school. The application of the protocol reduced the indoor/outdoor (I/O) ratio for all toxic pollutants. The reduction was also remarkable for PM_10_—the most critical air quality parameter in the study area (1.5 and 1.1 for non-alert and alert days, respectively). Indoor concentrations of PM_10_—especially during non-alert days—were often higher than outdoors, showing a major contribution from resuspension due to the movement of people and personal cloud. The protocol did not cause any increase in indoor CO_2_ levels. Our findings showed that the application of a ventilation protocol together with the contribution of an air purifier may represent an effective way to reduce children’s exposure to air pollution during severe air pollution episodes. Considering the onset of COVID-19 and the airborne transmission of pathogens, this protocol now has more meaningful implications for children’s welfare, and can be integrated with protocols designed as measures against the spread of SARS-CoV-2.

## 1. Introduction

Exposure to air pollution has been associated with both short- and long-term health effects [1,2]. Children in particular are considered to be vulnerable subjects, and in young populations air pollution can trigger asthma symptoms and impact on neurodevelopment and cognitive ability [3].

People spend more than 90% of their time indoors, where the majority of exposure to air pollutants occurs. As a matter of fact, indoor exposure is by far the main contributor to total personal exposure in relation both to indoor- and outdoor-generated air toxins. Building envelopes represent a protection against the pollutants coming from outdoors, and without indoor sources indoor concentrations would be lower than those outdoors [4]. However, indoor sources may significantly contribute to indoor concentrations and levels of some pollutants, even in residential settings, which may become far greater than those found outdoors [5].

School is an indoor environment that deserves particular attention, because exposure in schools accounts for a significant quota of the daily exposure faced by children [6]. While no type of combustion is present inside schools and classrooms, air quality can be worsened by the high number of people gathering in small spaces with low air-exchange rates. Several studies have shown the importance of resuspension and personal cloud in increasing indoor concentrations. The mere presence of people in indoor environments is associated with increase in particle concentration, and this is due to both exogenous (i.e., not emitted by the human body envelope) and endogenous sources [7].

Exogenous sources include activities not present in schools, such as cooking and smoking, but also school-related contributions such as vacuuming and resuspension from flooring due to walking, running, and exercising [8,9,10].

Endogenous sources include human skin fragments and airborne particles generated through frictional interaction with clothing fibers [11]. Previous studies have documented a strong link between indoor coarse particle concentrations and occupancy-associated emissions through body envelope shedding [12,13]. A relevant contribution to indoor PM levels from handling office paper and clothing fabric was observed in various studies [14,15]. It is worth noting that clothing can also contain microorganisms such as bacteria and viruses [16,17] that, together with bacteria-laden skin flakes, fragments, and fibers, can be dispersed to the surroundings via occupants’ activities [18].

Among endogenous sources, a primary role is also played by bioaerosols produced while breathing and speaking. This contribution is very important for viral and bacterial transmission, but is not relevant for increasing particulate matter concentrations [19].

Another relevant impact on indoor air quality inside school buildings is related to cleaning activities [20,21]. The extensive use of cleaning products indoors leads to the formation of fine particles (PM_1_ and UFPs), with various possible consequences for children’s health.

A number of studies have evaluated the concentration levels of different pollutants in classrooms [22]. Carbon dioxide (CO_2_) has often been considered a good proxy for indoor air quality [23,24]. High concentrations of CO_2_ itself have been associated with negative effects on students’ vigilance and attention [25,26].

More severe health effects on children are caused by other pollutants, such as nitrogen dioxide (NO_2_) and particulate matter. NO_2_ is mainly generated by combustion sources, and can cause respiratory effects such as increased inflammation of the airways, worsened cough and wheezing, reduced lung function, and increased asthma attacks [2]. Thus, no indoor sources of NO_2_ are present in schools, and indoor concentrations derive from penetration from outdoors. Even more detrimental effects are associated with particulate matter (PM) exposure, usually considered in terms of exposure to PM_10_ (i.e., airborne particles with diameters below 10 µm), PM_2.5_ (i.e., particles below 2.5 µm), and PM_1_ (i.e., particles below 1 µm). Respiratory, cardiovascular, neurodegenerative, and carcinogenic effects have been documented in a number of studies [27,28,29]. No proper primary sources of PM are generally present in schools, but resuspension, human skin and clothing, and furniture can lead to elevated concentrations.

Due to the substantial portion of time spent by children in school settings, indoor air quality in schools is particularly relevant, and prevention actions aiming at reducing pollutants’ concentrations and health effects are of primary importance. The recent COVID-19 pandemic has further underlined how important indoor conditions and air circulation are for health and wellbeing [30].

A first field of intervention concerns increasing communication and awareness. Several studies [31,32,33,34,35] have tried to assess the impact of timely and personalized communication highlighting the effectiveness of paying attention and changing personal habits in case of bad air quality. To this end, international bodies as well as research groups have tried to define specific indications in relation to the different air quality levels, while also considering synthetic indicators such as air quality indices (e.g., Air Parif, https://www.airparif.asso.fr/en, accessed on 30 August 2022; Air Now, https://www.airnow.gov, accessed on 30 August 2022).

Equally or even more important to prevent negative effects on children’s health are the actions aiming at improving indoor air quality in schools. While a number of studies have assessed the air quality in schools, only a few have tried to assess the effectiveness of specific interventions. Considering school buildings without heating, ventilation, and air conditioning (HVAC) systems—i.e., by far the majority of such buildings in the world—and without considering changes in the structural characteristics of the buildings [36] and furniture, the possibilities to improve indoor air quality are mainly related to opening windows and doors, and to the possible use of portable air purifiers.

Several studies [37,38] have shown the differential impacts of opening windows on indoor- and outdoor-generated pollutants. Increased airing was associated with reduced concentrations of CO_2_ and volatile organic compounds (VOCs), and elevated values of NO_2_ and polycyclic aromatic hydrocarbons (PAHs), showing a tradeoff often required in setting up strategies to reduce indoor- and outdoor-generated pollutants. Peculiar findings were reported for PM exposure, with smaller fractions (ultrafine particles—i.e., particles below 100 nm—and PM_1_) showing reduced concentrations associated with increased airing, while PM_10_ and, to a lesser extent, PM_2.5_ showed behaviors more similar to indoor-generated pollutants. Especially in school environments, the benefits related to the use of air purifiers have been receiving increased attention. Under proper use and circumstances, it seems that these devices are able to significantly improve indoor conditions [39].

In this study, we tested a protocol designed to reduce children’s exposure to air pollution during alert days. The focus was on NO_2_ and three PM size fractions (i.e., PM_1_, PM_2.5_, and PM_10_). The underlying idea was that during alert days the priority should be the reduction in the concentrations of outdoor-generated pollutants, and that the application of the protocol being limited to specific days could constitute a reasonable commitment for students and teachers. The secondary goal was to verify the practical feasibility of a prompt and timely communication chain to alert school personnel and activate the protocol on alert days. To the best of our knowledge, this is the first study to test the impact of a preventive measure aiming at reducing children’s exposure during air pollution episodes.

## 2. Materials and Methods

### 2.1. Settings

The monitoring campaign was conducted at the “Cocconi” primary school (Istituto Comprensivo Parmigianino). The school is located in the urban area of Parma (Italy)—a city of about 196,000 inhabitants (2021). The municipal territory is in the southern part of the Po Plain, Northern Italy, and is affected by high levels of air pollution [40]. The building that houses the school (P.le Picelli 3, Parma) is located near the historic center of the city, and was built in the early 1900s. The building has undergone numerous renovations over the past years. The location of the classroom is on the first floor, overlooking a pedestrianized street. The dimensions of the classroom are approximately 8 m × 10 m, with high ceilings (4 m) and 3 large windows. The floor is marble, the paint used for the walls and ceiling is water-based, and the furniture is made of wood and metal. The classroom accommodates 24 children.

### 2.2. Alert System

The alert procedure was based on the air quality forecasts provided by the Environmental Agency of the Emilia-Romagna Region (Arpae). The model suite is called NINFA, and includes a chemical transport model called CHIMERE, a national numerical weather prediction model called COSMO, and a statistical post-processing module called IBIS.

CHIMERE (https://www.lmd.polytechnique.fr/chimere/, accessed on 30 August 2022) is the core component of the NINFA modelling system. CHIMERE is an open-access multiscale Eulerian chemistry transport model that simulates the transport, dispersion, chemical transformations, and deposition (dry and wet) of air pollutants and aerosols.

The COSMO model (http://www.cosmo-model.org, accessed on 30 August 2022) is the official Italian limited-area atmospheric model, which is also used at the Italian National Civil Protection Department. It is a non-hydrostatic limited-area atmospheric prediction model based on primitive thermo-hydrodynamic equations describing compressible flow in a moist atmosphere, with a variety of physical processes taken into account by parameterization schemes. In the air quality system, the COSMO model operates in two main configurations: one with a 5 km grid spacing and 45 vertical levels over the Mediterranean area (COSMO-5M), and one with a 2.2 km grid spacing and 65 vertical levels over Italy (COSMO-2I).

NINFA is run by Arpae Emilia-Romagna in a nested configuration. In particular, the nesting chain includes a run at 5 km resolution over Northern Italy and a run at 3 km resolution focused on the Emilia-Romagna region. The concentration fields are post-processed by a statistical Bayesian model called IBIS (Inferenza Bayesiana Inquinamento Simulato). The IBIS model uses time series of measured data to correct the model outputs and produce probabilistic predictions for the same day and the next two days.

When the AQ forecasting model predicted the exceedance of the daily PM_10_ concentration for the next day, this triggered the dispatch of information to teachers about the protocol (see Section 2.3).

### 2.3. Definition of the Protocol

The ventilation protocol foresees that the classroom door should always be kept open, while the windows should be open only during the mid-morning break. In addition, an air purifier should be activated. The underlying criterion was to try to limit the opening of windows to reduce air exchange between indoors and outdoors and, consequently, to reduce the entry of pollutants from outdoors. The door being kept open is intended to prevent CO_2_ concentrations from becoming too high.

Teachers and students were asked to fill in a daily report where the window-opening hours were indicated together with blank spaces to report some specific events (e.g., students out of the classroom, no regular lessons, etc.). No data about window opening during non-alert days were available.

Teachers and students were asked to apply the protocols in the event of alert days. Alert days are considered to be those days characterized by forecasted PM_10_ concentrations above the daily limit (50 µg/m^3^). The forecasts were performed one day in advance and delivered to the teaching staff during the afternoon of the same day (i.e., the day before the day classified as an alert day). Alert days were communicated via smartphone (by the creation of a WhatsApp^®^ group). On non-alert days, no ventilation recommendations were provided to students and teachers.

### 2.4. Instruments and Measurement Campaign

**Figure 1:**
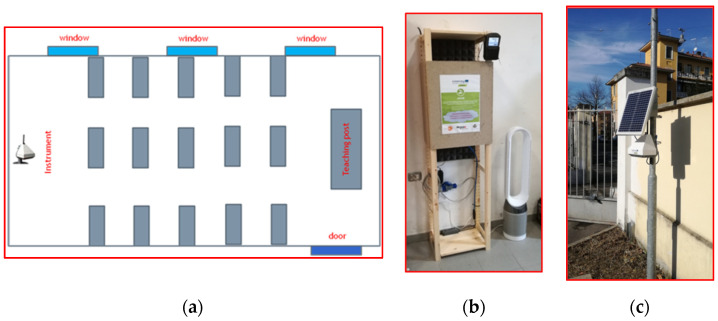
Location of the AQMesh measuring instrument in the classroom (**a**), support structure for AQMesh and air purifier (**b**), and location of the outdoor instrument with its solar panel (**c**).

The monitoring campaigns were conducted with two units (pods) of a sensor-based system called AQMesh. AQMesh pods (Environmental Instruments Ltd., Stratford-upon-Avon, UK) are portable, small (22 cm × 16 cm × 20 cm), and lightweight (<2 kg) instruments equipped with an electrochemical NO_2_ sensor (Alphasense NO2-B43F), an infrared CO_2_ sensor (Alphasense IRC-A1), an OPC (optical particle counter) sensor for the measurement of PM_1_, PM_2.5_, and PM_10_, and three solid-state sensors for temperature (T), relative humidity (RH), and atmospheric pressure (P). One sensor system was placed indoors in the school classroom, while the other was placed outdoors near the school (Figure 1c). The indoor system was powered by the electricity grid, while the outdoor system was powered by a solar panel. For easier placement of the instruments inside the classrooms, three structures were prepared. Due to the slight but perceptible noise due to the suction pump connected to the OPC sensor, the structures were equipped with sound-absorbing panels. The indoor pod was placed at the back of the classroom. Figure 1 shows the layout of the classroom, the placement of the instruments together with their support structure, and the instrument placed outdoors together with its solar panel.

An air purifier (Dyson^®^ Pure Cool) was placed at the back of the classroom (Figure 1a).

The monitoring campaign in the school lasted from 9 January 2020 to 11 February 2020. The choice of the winter period for the monitoring campaign was because this is the period when almost all of the alert days occur in the area. Some problems affected the monitoring campaign. The first critical issue concerned the interruption of the operation of the indoor pod from 30 January to 4 February. Some problems also affected the pod placed outdoors, which sometimes showed deliquescence problems related to high relative humidity. These issues led to a reduction in the number of days available for the analyses; in total, 6 alert days and 7 non-alert days were ultimately considered.

## 3. Results

### 3.1. Characteristics of the Monitoring Period and Effectiveness of the Forecasting System

**Figure 2:**
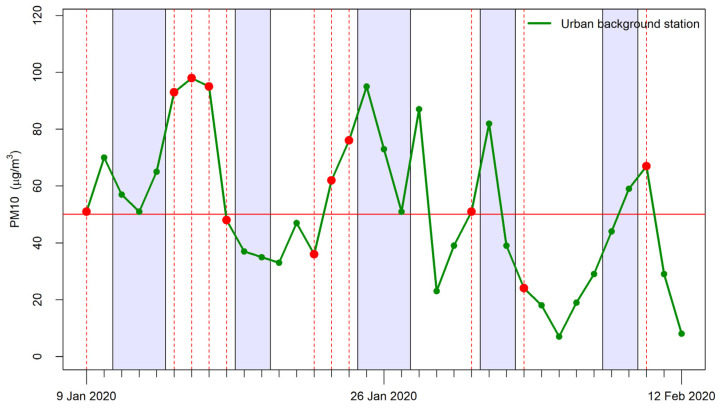
Daily mean PM_10_ concentration measured by the urban background station during the monitoring campaign. Red circles indicate alert days. The red line represents the EU daily limit value (50 µg/m^3^).

The monitoring period was characterized by frequent alert days. Table 1 shows some descriptive statistics for PM_10_, PM_2.5_, and NO_2_ concentrations as measured by the reference urban background monitoring station located about 1.5 km from the school. Figure 2 shows the daily mean PM_10_ trend over time during the period, as measured by the same station. The figure also shows the alert days as delivered by the Arpae air quality forecasting system. The data show that, considering only school days, 8 of the 10 exceedances of the limit value of PM_10_ were correctly predicted. Three alerts were false alarms. To assess the effectiveness of the protocol, we selected only correctly forecasted alert days.

### 3.2. Indoor and Outdoor Mean Pollutant Concentrations

Table 2 shows an overview of the mean indoor and outdoor concentrations of the measured pollutants for alert and non-alert days. The data considered refer to the morning school hours (8:30–12:30 a.m.), and all of the subsequent analyses refer to the data measured in the same time window. The sampling frequency of the sensors was 15 min.

Outdoor concentrations of NO_2_ and PM were considerably higher during alert days compared to non-alert days. The greatest difference (56.9 vs. 18.3 µg/m^3^) was found for PM_1_, the concentrations of which were almost entirely due to combustion sources and chemical reactions in the air (i.e., “secondary particulate”). The mean outdoor PM_10_ concentration during school hours on alert days was 44.9 µg/m^3^—lower than the mean daily concentration on alert days (53 µg/m^3^). This is due to the typical daily temporal pattern of PM_10_ in the area [41]. Conversely, indoor concentrations were similar between alert and non-alert days. The mean PM_10_ concentrations during school hours on alert days and non-alert days were 48.1 and 49.3 µg/m^3^, respectively—both lower than the EU daily limit value. The mean indoor CO_2_ concentrations were similar between alert and non-alert days (2446.7 and 2389.1, respectively), and well above the ASHRAE guideline values (1000 ppm) [42]. Hourly values of about 3500 ppm were frequently observed towards the end of the morning school hours, with concentration peaks of 4500 ppm.

### 3.3. Indoor and Outdoor Time Trends

**Figure 3:**
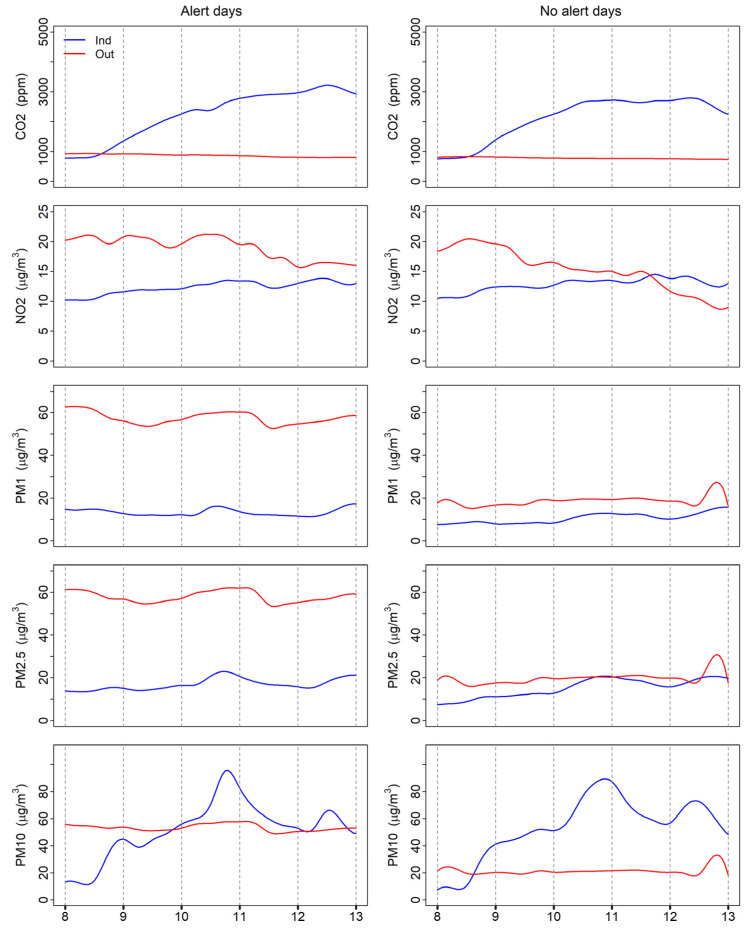
Mean trends of measured pollutants during school hours. The blue lines are the indoor concentrations, while the red lines are the outdoor concentrations, as measured by the AQMesh sensors.

A uniform increasing trend during school hours was observed for CO_2_, especially for alert days (Figure 3). During non-alert days the curve flattened in the second part of the morning, when manual airing usually intensifies.

As expected on the basis of the daily evolution of the planetary boundary layer height and the emission of traffic-related pollutants, outdoor NO_2_ concentrations showed a marked decrease during the morning [41]—especially during non-alert days. Conversely, indoor NO_2_ concentrations showed a slightly increasing trend. Due to the time lag of outdoor-to-indoor pollutant penetration, higher NO_2_ concentrations were found indoors compared to outdoors at the end of the school hours during non-alert days.

While almost constant concentrations of PM_1_ and PM_2.5_ were measured both indoors and outdoors, a different behavior was observed for coarser particles. Indoor PM_10_ levels showed a marked increase in concentration during the early morning hours, with a peak during the mid-morning break (from 10.30 to 10.45 a.m.). Afterwards, the indoor PM_10_ concentrations showed a decreasing trend, probably due to the opening of the windows. A final secondary peak was observed for indoor PM_10_ at the end of the lessons (12.30 a.m.). Very similar trends were observed during alert and non-alert days. Conversely, outdoor PM_10_ concentrations remained almost constant. Small peaks in outdoor concentrations were observed at the beginning and the end of the morning of the non-alert days, probably due to the impact of increased traffic congestion around the school.

### 3.4. Effectiveness of the Application of the Protocol

The effectiveness of the application of the protocol was assessed in terms of the comparison of the ratio between indoor and outdoor concentrations of the pollutants (I/O ratio).

**Figure 4:**
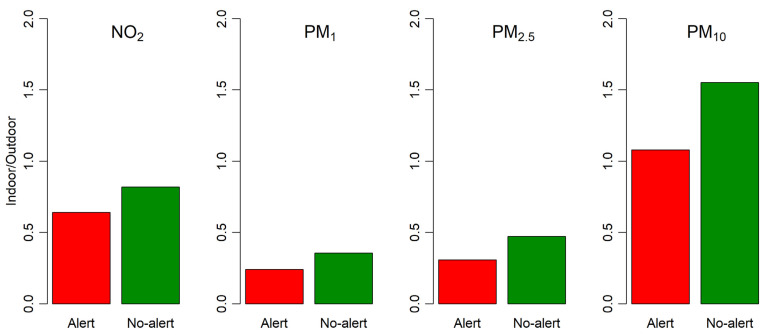
Comparison of the indoor/outdoor ratios of the measured toxic pollutants during alert and non-alert days.

All pollutants showed lower I/O ratios on alert days compared to non-alert days (Figure 4). The I/O ratios were lower than 1 for all pollutants except for PM_10_, which showed I/O ratios equal to 1.1 and 1.5 for alert and non-alert days, respectively. PM_10_ was also the pollutant with the highest absolute difference between I/O ratios on alert and non-alert days.

## 4. Discussion

The results showed that PM_10_ concentrations were often higher indoors than outdoor, especially during the mid-morning break and the second half of the morning. The elevated indoor PM_10_ concentrations were the result of the movements and activities of children and teachers.

Our data highlight the importance of the contribution of the presence of people in schools in causing elevated coarse particle concentrations. In particular, the increasing impact of resuspension phenomena during the morning was documented, along with a sharp peak in coarse particle emissions during the mid-morning break. These results should be considered in defining strategies to reduce children’s exposure as well as in the exposure assessment phases of epidemiological studies involving schoolchildren.

In agreement with previous findings [43], no marked effects of the presence of people and activities were observed for PM_1_ or PM_2.5_.

As for the effect of cleaning activities inside the school building, we believe that the impact on the measuring campaign was substantially negligible. In fact, cleaning is carried out during the afternoon hours, while only the school morning hours (8:30–12:30) were considered in our analysis.

The application of the protocol was effective in decreasing the exposure of children to pollutants. The I/O ratios were lower on alert days compared to non-alert days for all pollutants. It should be noted that the effectiveness was high with regards to all pollutants, including PM_10_, which is the most critical pollutant in the study area, and was the air quality parameter used to classify the alert and non-alert days. To the best of our knowledge, this is the first study testing the effectiveness of a protocol aiming at reducing children’s exposure to air pollutants in schools. Numerous studies have investigated the role of manual airing, vacuuming, and mechanical ventilation in determining the indoor concentrations of pollutants, but to the best of our knowledge no previous study has tested the practical application of a protocol. Two main features were considered in defining the protocol: First, the ease of application; feedback from teachers and students was positive in this sense, and it seemed that the frequency of window opening and activation of the air purifier could be managed with reasonable commitment. Second, the limitation of the application of the protocol to the forecasted severe air pollution episodes; the good performance of the air quality forecasting system enabled us to use it as a key component of a prevention system, and to limit the frequency of application of the protocol. It is worth noting that the tradeoff between indoor- and outdoor-generated pollution (including resuspension and personal cloud contribution) probably prevents the definition of an effective protocol for all environmental conditions. As a matter of fact, the frequency of manual airing should be increased as much as possible when outdoor pollutant concentrations are low, and limited in the event of severe air pollution episodes.

The indication of keeping the classroom’s door open during the alert days deserves a brief discussion. The aim of this indication was to prevent indoor CO_2_ concentrations from increasing excessively because of a reduced air-exchange rate. Our findings showed that this goal was achieved, with very similar mean CO_2_ concentrations and trends between alert and non-alert days. However, it should be noted that the CO_2_ concentrations were considerably high (over 2000 ppm), and could have an impact on the performance of the children.

Our study was not designed to disentangle the specific contributions of the air purifier and the opening of the windows and door. However, based on previous findings, it is reasonable to posit that the air purifier is especially effective in removing larger particles (i.e., PM_2.5_ and PM_10_). Oh et al. [44] documented an 86% and 69% decrease in PM_2.5_ and PM_10_ concentrations, respectively, in childcare centers in Korea. Fermo et al. [45] observed a 90% and 80% descrease in PM_10_ and PM_2.5_ concentrations, respectively, in a small room of an apartment after the activation of an air purifier. A similar decrease in concentrations of PM_1-10_ after activation of a purifier was observed by Pacitto et al. [38] in two school gyms under natural ventilation conditions. However, much smaller effects were reported by the authors under manual airing conditions. A substantial (about 35%) but lower decrease in indoor exposure to coarse particles associated with the use of an air purifier was also reported by Park et al. [46] in a study involving 34 elementary schools in Korea. Our data suggest an impact of the air purifier in the classroom no greater than 30% in magnitude. It seems that the opening of windows three times during the morning and the frequent movements of children during the school hours reduced the effectiveness of the air purifier in filtering airborne particles.

The monitoring campaign in the school was included in the activities of the AWAIR Interreg project, and was accompanied by educational activities aiming at increasing awareness and showing children how to collect information on air quality from official sources. The experimental and educational activities have laid the groundwork for the presentation of the smartphone app “Interreg SAPEs app”, which allows children to know the expected levels of pollution and the possible risks every day, to receive tips and, in case of severe pollution episodes, to apply the protocol.

## 5. Conclusions

The application of a protocol based on regulation of manual airing combined with the activation of an air purifier was effective in reducing children’s exposure to air pollutants in a classroom during severe air pollution episodes. The protocol was designed to be easy to apply, and was combined with an air quality forecasting system that enables its application only on alert days. Its use may be recommended in all schools lacking in mechanical ventilation and filtration systems, and especially in classrooms where vulnerable children are present.

## Figures and Tables

**Table 1:** Summary statistics of mean urban environmental conditions during the monitoring period: Air pollutant data are from the urban background reference station “Cittadella”, and meteorological data are from a reference urban meteorological station.

	PM_10_ (µg/m^3^)	PM_2.5_ (µg/m^3^)	NO_2_ (µg/m^3^)	Temp °C	RH %
Mean	53	35	38	6.6	74
Min	7	5	8	−2.7	12
Max	98	82	85	19.0	101

**Table 2:** Mean concentrations of selected pollutants as measured indoors and outdoors during school hours.

		CO_2_ (ppm)	NO_2_ (µg/m^3^)	PM_1_ (µg/m^3^)	PM_2.5_ (µg/m^3^)	PM_10_ (µg/m^3^)
Alert days						
	Indoor	2446.7	12.5	13.2	17.2	48.1
	Outdoor	936.1	20.8	56.9	47.9	44.9
Non-alert days						
	Indoor	2389.1	13.2	10.6	15.8	49.3
	Outdoor	833.3	16.8	18.3	18.4	19.4

## Data Availability

Not applicable.
